# The impact of social support on the quality of life among older adults in China: An empirical study based on the 2020 CFPS

**DOI:** 10.3389/fpubh.2022.914707

**Published:** 2022-09-07

**Authors:** Tongtong Shen, Dongju Li, Zengyun Hu, Jie Li, Xi Wei

**Affiliations:** ^1^School of Statistics, Dongbei University of Finance and Economics, Dalian, China; ^2^School of Statistics and Big Data, Henan University of Economics and Law, Zhengzhou, China; ^3^School of Economics, Henan University, Kaifeng, China; ^4^State Key Laboratory of Desert and Oasis Ecology, Xinjiang Institute of Ecology and Geography, Chinese Academy of Sciences, Urumqi, China

**Keywords:** older adults, quality of life, formal social support, informal social support, Shapley value decomposition

## Abstract

**Background:**

As aging issues become serious, how to guarantee and improve the quality of life among older adults has become a hot topic in China. This article is aimed to discuss the impact of formal and informal social support on the quality of life among older adults and the differences in gender and urban–rural areas.

**Methods:**

The data used in this article are from the 2020 China Family Panel Studies (CFPS). Quality of life is measured from three dimensions of life: satisfaction, self-rated health, and mental state. This article uses the ordered logistic regression model to analyze the impact of social support on life satisfaction and self-rated health, and the binary logistic regression model to analyze the impact of social support on the mental state. The method of Shapley value decomposition further analyzes the contribution of influencing factors to the quality of life.

**Results:**

The activities of daily living (ADL) and income significantly impact the quality of life among older adults. Formal and informal social support positively improved the quality of life among older adults, but the effect of informal social support is greater than that of formal social support. The male older adults are significantly better than the female adults across all three dimensions of quality of life. The mental state of urban older adults is better than that of rural older adults.

**Conclusion:**

Formal and informal social support should be strengthened to improve the income of older adults. Older adults should be encouraged to participate in social activities and good interpersonal relationships should be established actively. Female older adults should be paid more attention. The proportion of female older adults participating in insurance should be increased, and the family and intergenerational care burden for female older adults should be reduced. The leisure life of urban older adults should be enriched. The basic social insurance and health service systems in rural areas should be improved.

## Introduction

Aging has been accelerating, and the population has shown a trend of advanced age since China entered an aging society around 2000 (1). According to the Seventh National Population Census from the National Bureau of Statistics in China, the population aged 60 and above was 264.02 million in 2020, accounting for 18.70% of the total population, an increase of 5.44% points over 2010 and 8.37% points over 2000 (2, 3). The number of people aged 65 and above was 190.64 million in 2020, accounting for 13.5%, an increase of 4.63% points over 2010 and 6.54% points over 2000 (2, 3). It is expected that the population aged 65 and above will exceed 200 million in China by 2022, with an aging rate of over 14%, and China will enter a deeply aging society (4). The pressure on the governments and families is increasing in the face of severe aging. The pension problem has become an urgent social problem to be solved. Ensuring older adults enjoy their twilight years and have a healthy and decent old life has become one of the major social development issues.

To deal with the aging of the population actively, the World Health Organization has put forward the concept of healthy aging and active aging. The core is to improve the quality of life among older adults and guarantee their right to development. The State Council issued a guideline to promote the development of national undertakings for the aged and improve the older adults' care service system during the 14th Five-Year Plan period (2021–2025) in China. The guideline proposes incorporating the concept of active aging and healthy aging into the economic and social development process to meet the need for high-quality services for the elder (5). Quality of life is one of the important indicators reflecting the living conditions of older adults, which is the basis for implementing healthy aging and active aging strategies. There are many factors influencing the quality of life among older adults, such as income status, education attainment, marriage, and living conditions. Social support also plays an important role in the improvement of the social security system.

## Literature review

The concept of quality of life is dynamic, complex, and multidimensional. This concept first appeared in the book The Affluent Society, written by American economist John Kenneth Galbraith. The author believes the quality of life is a subjective experience in nature, including personal satisfaction with life experience, internal sense of contentment, and self-realization in society (6). Later scholars gradually crystallized the quality of life and understood it from multiple dimensions but failed to form a unified definition. The first understanding is to define the quality of life as a comprehensive reflection of objective living conditions, such as the area of the living house (7). The second understanding is to regard the quality of life as a subjective feeling of the overall life, such as life satisfaction and other subjective evaluation indicators (8, 9). The third understanding is to combine the objective part and the subjective part. The quality of life comprises two parts: the objective conditions reflecting living conditions and subjective feelings about the living conditions (10, 11). For older adults as a special group, a lot of literature defines and measures the quality of life among older adults according to research objectives. The quality of life among older adults proposed by the Chinese Medical Association in 1994 includes 11 aspects: health status, living habits, functions of daily living, family harmony, living conditions, economic income, nutritional status, mental health, social interaction, life satisfaction, and physical examination (12). Wu believed that the quality of life among older adults includes material life, spiritual and cultural life, life quality, personal quality, rights and interests, and living environment (13). After analyzing 48 studies on older adults, Van Leeuwen et al. determined that the quality of life among older adults should include nine domains: autonomy, role and activity, health perception, relationships, attitude and adaptation, emotional comfort, spirituality, home and neighborhood, and financial security (14). Due to the availability of survey data, this article refers to research of Li and determines three dimensions of the quality of life among older adults: life satisfaction, self-rated health, and mental state (1).

The concept of social support was first developed as a technical term in psychiatric literature. It is defined as information leading one to believe that he/she is cared for, loved, esteemed, and a member of a network of mutual obligations (15). Subsequently, many scholars have extensively researched social support as a science, and there are many definitions of social support. In general, the definition of social support can be viewed in four ways. Social support, which can be generated from helping behavioral, is a kind of interpersonal interaction, an exchange of social resources, and a systematic psychological activity (16). Additionally, there are a variety of classifications of social support. According to the functions of social support, Flannery categorized emotional support, instrumental support, informational support, and social companionship (17). Mindel et al. categorized formal and informal social support according to the support subjects (18). Formal social support refers to the support provided by the government, institutions, communities, and other formal organizations for vulnerable groups, such as endowment insurance and the medical security system (19). Informal social support refers to the emotional, behavioral, and informational support provided by family members, neighbors, friends, and colleagues (16).

Developed countries were the first to study the impact of social support on quality of life. The results showed that social support positively impacted the quality of life and improved health and mental status (20–22). Social support has been found to play an important role in the quality of life in the literature with studies of Chinese older adults (23, 24). Various types of formal social support have been shown to positively affect older adults' life satisfaction and physical and mental health. Tao and Shen found that the new rural endowment insurance and rural medical insurance have a positive impact on the mental health of rural older adults but little contributes to their physical health (16). Zheng and Zheng found that the basic endowment insurance for the urban working group can improve the life satisfaction of older adults, while participation in the new rural endowment insurance system and new cooperative medical system will significantly improve the intergenerational financial support for families to indirectly promote the health and life satisfaction of older adults (25). Deng and Tang found that participating in endowment insurance positively impacts the life satisfaction of older adults (26). Li et al. found that older adults participating in endowment insurance had better self-rated health and that endowment insurance, medical insurance, and other social assistance significantly affected the degree of depression (27).

Regarding informal support, it is usually the impact of factors, such as intergenerational support and family support on the quality of life among older adults. Li found that practical, emotional, and spiritual social support positively affected older adults' life satisfaction, health status, and mental health (1). Wei et al. found that intergenerational support and social interaction can reduce the loneliness of rural older women and promote their physical and mental health (28). Li analyzed the impact of social support (emotional support, economic support, and daily care) on the quality of life among older adults and found that all social support variables positively impacted the quality of life (29). Fu and Cheng found a significant contribution of intergenerational support to older adults' life satisfaction (30).

**Figure 1:**
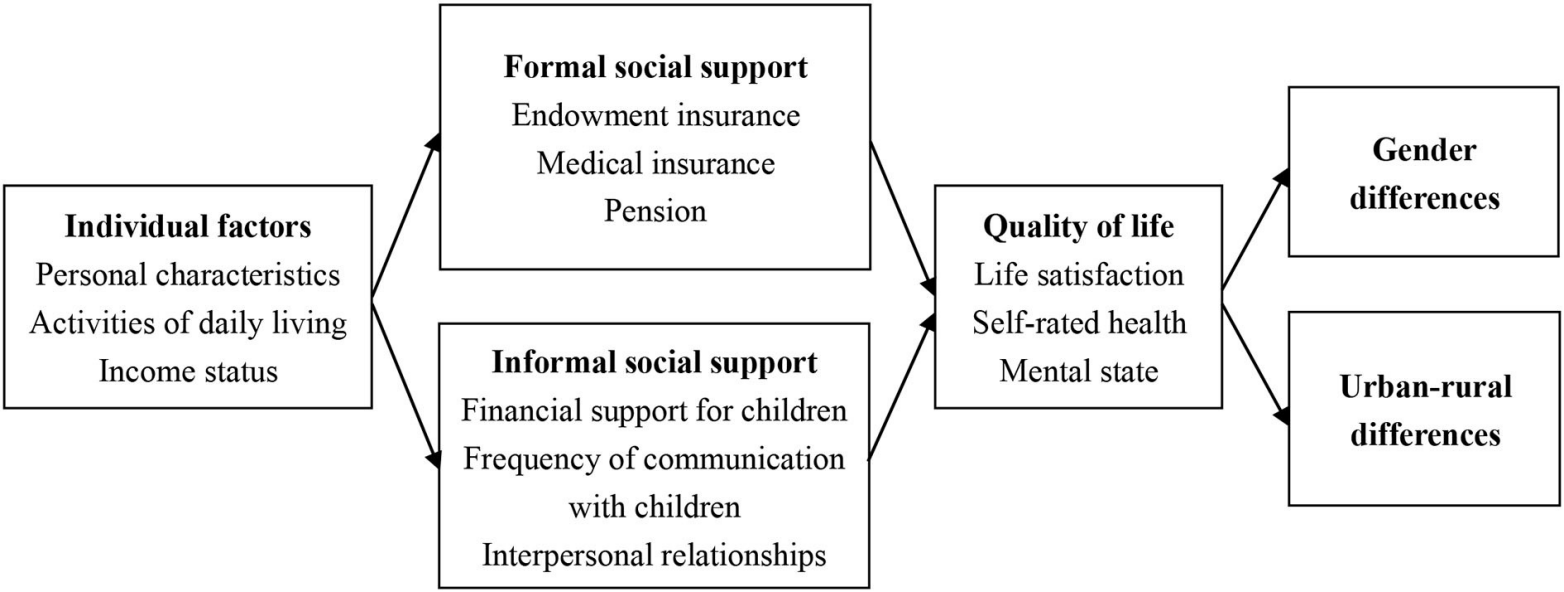
Research framework.

To sum up, this article analyzes the impact of formal and informal social support on the quality of life (life satisfaction, self-rated health, and mental state) among older adults. The influence of activities of daily living and income status on the quality of life is also considered because a good quality of life for older adults is based on a certain material basis and independent activity ability (31). The research framework of this article is shown in Figure 1. This article lies in the comprehensive analysis of the impact of social support on the different aspects of quality of life for older adults. It further analyzes the difference of this impact in different groups of older adults, which is helpful to put forward targeted suggestions on the pension cause and better achieve a balanced pension.

## Materials and methods

### Data sources

The data come from the 2020 China Family Panel Students (CFPS), conducted by the Institute of Social Science Survey of Peking University. The adult questionnaire includes questions on basic personal information, retirement, insurance, marriage, and health status. The research topic of this article is the impact of social support on the quality of life among older adults. First, 6,976 older adults aged 60 and above were screened from 28,590 adult samples. Second, 1,847 older adults answered the questionnaire on behalf of others without answering subjective questions, such as life satisfaction and income status. Another 1,268 older adults did not answer questions about the physical test due to telephone interviews. Then, some samples with missing variables, such as whether they have a pension, were removed. Finally, 3,323 older adults with complete variable values were obtained after elimination.

### Research variables

#### Explained variable

This article measures the quality of life among older adults from life satisfaction, self-rated health, and mental state, considering these three dimensions as explanatory variables. Life satisfaction was measured using a question in subjective attitude section, “How satisfied do you rate your life?” (30). Respondents were asked to rate their life satisfaction on a scale of 1–5, from 1 = being very dissatisfied to 5 = being very satisfied. Self-rated health was assessed using a question in the health section, “How do you feel about your health?” (1). Respondents could choose from five options, “Very good, Good, Fair, Poor, Very poor”. For the convenience of data analysis, these options were assigned a value from “Very poor” to “Very good” (Very poor = 1, Poor = 2, Fair = 3, Good = 4, Very good = 5). The mental state was measured by choosing the degree of depression. Center for Epidemiological Studies Depression Scale (CES-D) was used in the 2020 CFPS questionnaire to reflect the degree of depression. This article adopts the answer options of CES-D8 to calculate depression scores (32). CES-D8 is a simplified version of CES-D, asking respondents eight of the 20 questions from CES-D. The corresponding score is assigned according to the frequency of the occurrence of a specific emotion, “Hardly (<1 day)” = 0, “Sometimes (1–2 days)” = 1, “Often (3–4 days)” = 2, and “Most of the time (5–7 days)” = 3. CES-D8 has two positive and six negative indicators, of which the positive indicators are assigned inversely. The total score of CES-D8 is 24, with higher scores indicating higher levels of depression. The threshold for depression was set at 9 (32). The degree of depression was reassigned to 1 (no depression) for respondents with a score of <9 and 0 (depression) for respondents with scores of 9 or above.

#### Explanatory variables

Social support is used as the explanatory variable, divided into formal and informal social support. This article chose endowment insurance, medical insurance, and pension as the formal social support (19, 27). Informal social support is mainly measured by whether children provide financial support (16), the frequency of communication with children (26), and the interpersonal relationship.

#### Control variables

This article considers some personal characteristics, such as age, gender, educational background, marital status, and household registration, as control variables. The CFPS divides educational background into nine types. To facilitate analysis, they are combined into four types, i.e., no schooling (no schooling), primary school and below (illiterate and semiliterate, primary school), junior high school and high school (junior high school, high school/secondary school/technical school/vocational high school), junior college and above (college, bachelor, master, doctorate). In terms of marital status, “Unmarried,” “Divorced,” and “Widowed” were classified as living without a spouse or partner, while “Married” and “Cohabiting” were classified as living with a spouse or partner. This article selects seven questions in the physical test to measure the activities of daily living (ADL) (29), i.e., “Can you go outdoors/eat/do kitchen activities/use public transportation/go shopping/clean/do the laundry independently?”. Assign the answer “Yes” to 1 and “No” to 0. The scores of the seven questions were summed up as the variable value of ADL in this article. The range from 0 for “complete disability” to 7 for “complete self-care” reflects the level of ADL among older adults. Income status was measured using the question, “How would you rate your income in local area?” (30). Respondents were asked to rate their income on a scale of 1 (very low) to 5 (very high).

The description of explained, explanatory, and control variables is shown in Table 1.

**Table 1:** Variable definition.

**Type of variables**	**Categorical variables**	**Name**	**Descriptions and assignment**
Explained variable	Quality of life	Life satisfaction	A scale of 1–5 is from very dissatisfied to very satisfied
		Self-rated health	A scale of 1–5 is from unhealthy to very healthy
		Mental state	1 = No Depression, 0 = Depression
Explanatory variables	Formal social support	Endowment insurance	1 = Have, 0 = No
		Medical insurance	1 = Have, 0 = No
		Pension	1 = Have, 0 = No
	Informal social support	Financial support by children	1 = Have, 0 = No
		Frequency of communication with children	A scale of 1–7 is from low to high
		Interpersonal relationships	A scale of 0–10 is from very worse to very good
Control variables	Personal characteristics	Age	60 years old and above
		Gender	1 = Male, 0 = Female
		Marriage	1 = Live with a spouse or partner, 0 = Live without a spouse or partner
		Education	1 = No schooling
			2 = Primary school and below
			3 = Junior High School / High School
			4 = Junior college and above
		Household registration	1 = Urban, 0 = Rural
		Activities of daily living (ADL)	A scale of 0–7 is from complete disability to complete self-care
		Income	A scale of 1–5 is from very low to very high

### Model design

#### Ordered logistic regression model

The two variables of life satisfaction and self-rated health are ordered categorical variables, so the ordered logistic regression model was used to analyze the impact of social support on life satisfaction and self-rated health. The model is as follows:


$$\begin{array}{l} \ln\;\left[\frac{P\left(y\leq j|x\right)}{1-P\left(y\leq j|x\right)}\right]=\alpha_{j}+\overset{n}{\underset{i=1}{\sum }}\beta_{i}x_{i} \end{array}$$

where *y* represents life satisfaction and self-rated health, *j* represents the grade value of options (*j* = 1, 2, 3, 4, 5), *x*_*i*_ represents social support variables or control variables, *n* represents the number of explanatory variables, β represents a set of regression coefficients, and α is the intercept term.

#### Binary logistic regression model

The mental state is a binary variable. The binary logistic regression model was used to analyze the impact of social support on the mental state of older adults. The model is as follows:


$$\begin{array}{l} \ln\;\left[\frac{P\left(y=1|x\right)}{1-P\left(y=1|x\right)}\right]=\alpha +\overset{n}{\underset{i=1}{\sum }}\beta_{i}x_{i} \end{array}$$

where *y* is the mental state (the value of 1 indicates no depression), *x*_*i*_ represents social support variables or control variables, *n* represents the number of explanatory variables, β represents a set of regression coefficients, and α is the intercept term.

## Discussion

### Descriptive statistics

The selected older adults are between 60 and 95 years old, with an average age of 68.06. Regarding gender, 52.33% of older adults are male, and 47.67% are female, with a male to female ratio of 1.1:1. The gender distribution of the samples is balanced. Most of the older adults are married, accounting for 87.3%. The overall education level of older adults is relatively low. The older adults with primary school education and below accounted for 62.8%, while the older adults with junior college and above accounted for only 2.8%. Regarding urban–rural distribution, the proportion of the urban older adults is 47.19%, slightly less than that of the rural older adults. And the proportion of rural older adults is 52.81%.

### The impact of social support on life satisfaction

Table 2 shows the empirical results of the impact of social support on life satisfaction among older adults. Model 1a only considers the impact of control variables on life satisfaction, and the results show that gender, marital status, education level, ADL, and income significantly influence life satisfaction. The life satisfaction of male older adults is higher than that of female older adults. The life satisfaction of older adults without a spouse or partner is lower than that of older adults with a spouse or partner. Educational attainment had a negative effect on life satisfaction, i.e., the higher the educational attainment, the lower the life satisfaction. ADL also significantly negatively influences life satisfaction, which goes against our general perception that better activities of daily living are associated with higher life satisfaction. The negative effect may be explained by the fact that the seven activities reflect instrumental activities of daily living. Older adults with better ADL will continue to work or take care of family or grandchildren, which is detrimental to improving life satisfaction. Income has a positive effect on life satisfaction. Only a high income can guarantee daily life and contribute to the improvement of life satisfaction. Model 2a also considers the impact of formal and informal support on life satisfaction based on Model 1a. The results show that in terms of formal social support, endowment insurance and medical insurance positively impact the life satisfaction of older adults, indicating that having endowment insurance and medical insurance is conducive to improving the life satisfaction of older adults. Regarding informal social support, the results show that the frequency of communication with children and interpersonal relationships significantly positively impact the life satisfaction of older adults. Better emotional support is more likely to improve life satisfaction.

**Table 2:** The impact of formal and informal social support on life satisfaction.

**Variable**	**Model 1a**	**Model 2a**	**Model 3a Male**	**Model 4a Female**	**Model 5a Urban**	**Model 6a Rural**
**Control variable**						
Age	0.003 (1.003)	0.001 (1.001)	−0.003 (0.997)	0.007 (1.007)	0.004 (1.004)	−0.003 (0.997)
Gender	0.145** (1.156)	0.194*** (1.215)	–	–	0.305*** (1.357)	0.11 (1.117)
Marriage	0.193* (1.213)	0.191* (1.211)	0.243 (1.275)	0.164 (1.178)	0.156 (1.169)	0.238 (1.269)
Education	−0.321*** (0.726)	−0.358*** (0.699)	−0.349*** (0.705)	−0.384*** (0.681)	−0.305*** (0.737)	−0.459*** (0.632)
Household registration	0.05 (1.05)	0.088 (1.092)	0.226** (1.253)	−0.059 (0.942)	–	–
ADL	−0.058* (0.944)	−0.076** (0.927)	−0.061 (0.941)	−0.089** (0.915)	−0.11** (0.896)	−0.058 (0.944)
Income	0.668*** (1.949)	0.63*** (1.877)	0.603*** (1.828)	0.659*** (1.933)	0.612*** (1.844)	0.65*** (1.916)
**Formal social support**						
Endowment insurance		0.244** (1.276)	0.528*** (1.695)	−0.127 (0.88)	0.231* (1.26)	0.347 (1.415)
Medical insurance		0.216* (1.241)	0.313* (1.368)	0.14 (1.15)	0.138 (1.148)	0.325* (1.384)
Pension		−0.166 (0.847)	−0.39 (0.677)	0.152 (1.165)	−0.124 (0.884)	−0.268 (0.765)
**Informal social support**						
Financial support by children		0.088 (1.092)	0.095 (1.099)	0.073 (1.076)	−0.019 (0.981)	0.179* (1.196)
Frequency of communication with children		0.034* (1.035)	0.053** (1.054)	0.017 (1.017)	0.021 (1.021)	0.047* (1.048)
Interpersonal relationships		0.237*** (1.267)	0.251*** (1.286)	0.225*** (1.253)	0.264*** (1.303)	0.218*** (1.244)
*N*	3,323	3,323	1,739	1,584	1,568	1,755
LR chi2	487.39***	683.7***	340.48***	356.54***	330.63***	361.93***
Pseudo *R*^2^	0.0662	0.0929	0.0906	0.0991	0.0958	0.0929

***, **, and * were significant at 1, 5, and 10% levels, respectively. OR values are in parentheses.

Models 3a and 4a further show gender differences in life satisfaction among older adults. ADL has a significant negative effect on life satisfaction in female older adults but not in male older adults. Female older adults with better activities of daily living are more likely to take care of family or grandchildren. Trivial household work is not conducive to improving the life satisfaction of female older adults. Access to endowment and medical insurance can significantly improve the life satisfaction of male older adults, while these two formal social support variables do not affect the life satisfaction of women. Male older adults are more likely to work outside the home at a younger age, while female older adults are more likely to take care of their families. More men than women have access to endowment and medical insurance. In terms of informal social support, the frequency of communication with children has a significant positive impact on life satisfaction among male older adults, while it has no effect on female older adults. The reason may be that male older adults tend to be serious and uncommunicative, while female older adults have multiple options for confiding and communicating. The effect of emotional support from frequent communication with children was greater in men than in women.

Although model 2a shows no significant difference in the overall life satisfaction among older adults in urban and rural areas, model 5a and model 6a can further show the difference in influencing factors of life satisfaction between urban and rural older adults. ADL has a significant negative impact on the life satisfaction of urban older adults but has no impact on rural older adults. The urban older adults with better ADL have lower life satisfaction. Urban older adults face higher living costs than rural older adults and tend to continue working after retirement. Different insurances have different effects on the satisfaction among older adults in urban and rural areas. Endowment insurance can significantly improve the life satisfaction of urban older adults, while medical insurance is more important to the life satisfaction of rural older adults. In terms of informal social support, receiving financial support from their children and high frequency of communication with children can significantly improve the life satisfaction of rural older adults but has no effect on urban older adults. The reason may be that urban older adults have a higher pension than rural older adults and do not need financial support from their children. Urban older adults can participate in more leisure activities than the rural older adults after retirement. The way of emotional sustenance for urban older adults is also diverse.

### The impact of social support on self-rated health

Table 3 shows the results of the impact of social support on self-rated health among older adults. Model 1b only considers the influence of control variables on self-rated health, and the results show that gender, education level, ADL, and income significantly influence self-rated health. Male older adults are in better health than female older adults, and older adults with higher levels of education are in better health. Older adults with better activities of daily living have better health, which is consistent with the process of human aging. Older adults with higher incomes spend more on health care and have better health. Model 2b considers the impact of social support on self-rated health among older adults. None of the three variables of formal support significantly impacts self-rated health, indicating that these basic insurances have no significant effect on improving the health status of older adults. Regarding informal support, interpersonal relationships significantly positively impact self-rated health. Good interpersonal relationships can play an important role in maintaining older adults' physical and mental health.

**Table 3:** The impact of formal and informal social support on self-rated health.

**Variable**	**Model 1b**	**Model 2b**	**Model 3b Male**	**Model 4b Female**	**Model 5b Urban**	**Model 6b Rural**
**Control variable**						
Age	−0.003 (0.997)	−0.004 (0.996)	−0.013 (0.987)	0.007 (1.007)	−0.005 (0.995)	−0.001 (0.999)
Gender	0.29*** (1.336)	0.309*** (1.362)	–	–	0.371*** (1.449)	0.253*** (1.288)
Marriage	0.001 (1.001)	0.012 (1.012)	0.199 (1.22)	−0.06 (0.942)	−0.071 (0.932)	0.076 (1.079)
Education	0.172*** (1.187)	0.177*** (1.193)	0.126 (1.134)	0.258** (1.294)	0.188** (1.206)	0.18* (1.197)
Household registration	0.046 (1.047)	0.074 (1.077)	0.096 (1.101)	0.036 (1.037)	–	–
ADL	0.347*** (1.414)	0.346*** (1.414)	0.312*** (1.366)	0.389*** (1.476)	0.395*** (1.484)	0.314*** (1.369)
Income	0.274*** (1.316)	0.25*** (1.284)	0.258*** (1.295)	0.242*** (1.274)	0.204*** (1.226)	0.282*** (1.325)
**Formal social support**						
Endowment insurance		0.138 (1.147)	0.048 (1.049)	0.248 (1.281)	0.258** (1.294)	−0.131 (0.877)
Medical insurance		0.006 (1.006)	0.074 (1.077)	−0.042 (0.959)	−0.071 (0.932)	0.09 (1.094)
Pension		−0.127 (0.881)	−0.088 (0.916)	−0.18 (0.835)	−0.227 (0.797)	0.115 (1.122)
**Informal social support**						
Financial support by children		0.091 (1.095)	0.053 (1.054)	0.125 (1.133)	0.16 (1.173)	0.059 (1.061)
Frequency of communication with children		0.001 (1.001)	0.017 (1.017)	−0.013 (0.987)	0.005 (1.005)	0.001 (1)
Interpersonal relationship		0.099*** (1.104)	0.102*** (1.108)	0.0934*** (1.098)	0.125*** (1.134)	0.078*** (1.081)
*N*	3,323	3,323	1,739	1,584	1,568	1,755
LR chi2	305.65***	346.95***	161.7***	160.13***	166.06***	185.84***
Pseudo *R*^2^	0.0308	0.0349	0.0311	0.0342	0.0359	0.0353

^***^, ^**^, and ^*^ were significant at 1, 5, and 10% levels, respectively. OR values are in parentheses.

Models 3b and 4b further show the differences in self-rated health between male and female older adults. The three variables of formal support do not affect the self-rated health of male and female older adults. Interpersonal relationships significantly influence both male and female older adults in terms of informal support. Although there is no significant difference in self-rated health between urban and rural older adults, model 5b and model 6b further demonstrate the difference in influencing factors. Endowment insurance has a significant positive effect on the health status of urban older adults but has no effect on rural older adults. Endowment insurance can increase the disposable income of urban retired older adults, increasing health expenditure and improving health status.

### The impact of social support on the mental state

Table 4 shows the empirical results of the impact of social support on older adults' mental state. Model 1c shows that all control variables had significant positive effects on the mental state of older adults. The mental health of older adults becomes better as they get older. Male older adults are in better mental health than female older adults. The older adults with a spouse or partner have a better mental state than without a spouse or partner. The older adults with a higher education level have a better mental state. Urban older adults have better mental states than rural older adults. Older adults with better ADL and higher incomes have better mental health status. Model 2c further considers the influence of social support on the mental health of older adults. None of the three formal support variables significantly impact the mental state. More frequent communication with children and better interpersonal relationships are beneficial in improving mental state. Emotional support is more important than economic support for the mental health of older adults.

**Table 4:** The impact of formal and informal social support on mental state.

**Variable**	**Model 1c**	**Model 2c**	**Model 3c Male**	**Model 4c Female**	**Model 5c Urban**	**Model 6c Rural**
**Control variable**						
Age	0.021** (1.021)	0.021** (1.021)	0.025* (1.025)	0.018 (1.018)	0.02 (1.02)	0.02* (1.021)
Gender	0.517*** (1.677)	0.551*** (1.736)	–	–	0.635*** (1.887)	0.496*** (1.642)
Marriage	0.565*** (1.76)	0.564*** (1.758)	1.008*** (2.739)	0.357** (1.428)	0.676*** (1.965)	0.473*** (1.604)
Education	0.351*** (1.421)	0.323*** (1.381)	0.223* (1.249)	0.433*** (1.541)	0.397*** (1.487)	0.255* (1.291)
Household registration	0.477*** (1.611)	0.484*** (1.622)	0.6*** (1.822)	0.368*** (1.446)	–	–
ADL	0.223*** (1.249)	0.217*** (1.243)	0.147*** (1.158)	0.28*** (1.323)	0.22*** (1.246)	0.22*** (1.246)
Income	0.262*** (1.3)	0.234*** (1.264)	0.268*** (1.308)	0.213*** (1.237)	0.26*** (1.297)	0.219*** (1.245)
**Formal social support**						
Endowment insurance		−0.035 (0.965)	0.019 (1.019)	−0.043 (0.958)	0.001 (1.001)	−0.139 (0.87)
Medical insurance		0.129 (1.138)	0.281 (1.324)	0.015 (1.015)	0.083 (1.087)	0.187 (1.205)
Pension		0.045 (1.046)	−0.121 (0.886)	0.159 (1.172)	−0.133 (0.876)	0.224 (1.251)
**Informal social support**						
Financial support by children		0.11 (1.116)	−0.156 (0.856)	0.316** (1.371)	0.126 (1.134)	0.095 (1.1)
Frequency of communication with children		0.038* (1.039)	0.049 (1.05)	0.032 (1.033)	0.052 (1.054)	0.026 (1.026)
Interpersonal relationship		0.105*** (1.111)	0.118*** (1.125)	0.094*** (1.099)	0.155*** (1.168)	0.074***(1.077)
*N*	3,323	3,323	1,739	1,584	1,586	1,755
LR chi2	251.66***	284.66***	118.59***	126.88***	126.81***	123.65***
Pseudo *R*^2^	0.07	0.0792	0.0732	0.0664	0.0855	0.0598

***, **, and * were significant at 1, 5, and 10% levels, respectively. OR values are in parentheses.

Models 3c and 4c show gender differences in the effect of social support on mental state. The three variables of formal support still have no significant effect on the mental state of male and female older adults. In terms of informal support, receiving financial support from children has a significant positive effect on the mental state of female older adults but does not affect the mental state of male older adults. The reason may be that the income of female older adults is lower than that of male older adults. Receiving financial support from children can significantly improve the mental state of female older adults. Models 5c and 6c show the difference in the impact of social support on mental health between urban and rural older adults. The three variables of formal social support do not affect the mental health of urban and rural older adults. Only interpersonal relationships have a significant positive effect on mental health regarding informal social support.

### Shapley value decomposition of factors influencing quality of life among older adults

The method of Shapley value decomposition was first proposed by Shorrocks (33), which is used to compare the degree of impact of independent variables on dependent variables based on cooperative game theory. This article applies the Shapley value decomposition method to determine the contribution of explanatory variables to the quality of life among older adults. The results of Shapley value decomposition are shown in Table 5. For all older adults, income and interpersonal relationships are the main factors in improving life satisfaction, accounting for more than 90%. ADL and income are the main factors in improving health status, with a combined share of 72.44%. There is no absolute dominant influencing factor for the mental state. Comparatively, ADL, gender, and income are the key determinants, accounting for more than 15%, respectively. Overall, income status plays a vital role in three aspects of quality of life, and ADL plays a more critical role in self-rated health and mental state. For formal support, endowment insurance and medical insurance only play a weak role in improving life satisfaction. For informal support, interpersonal relationships considerably impact all three aspects of quality of life.

**Table 5:** Shapley value decomposition.

	**All**		**Male**		**Female**		**Urban**		**Rural**	
Life satisfaction	Income	61.28	Income	54.16	Income	67.08	Income	56.21	Income	65.44
	Interpersonal relationship	30.43	Interpersonal relationship	34.63	Interpersonal relationship	26.62	Interpersonal relationship	34.19	Interpersonal relationship	27.41
	Education	4.13	Education	4.14	Education	4.95	Education	3.85	Education	3.77
	Endowment insurance	0.86	Endowment insurance	2.62	ADL	1.35	Gender	2.44	Medical insurance	1.27
	ADL	0.84	Frequency of communication with children	2.02			ADL	1.92	Frequency of communication with children	1.17
	Frequency of communication with children	0.68	Medical insurance	1.94			Endowment insurance	1.39	Financial support by children	0.94
	Gender	0.64	Household registration	0.49						
	Marriage	0.57								
	Medical insurance	0.57								
Self-rated health	ADL	47.87	ADL	52.44	ADL	55.21	ADL	47.78	ADL	45.14
	Income	24.57	Income	29.66	Income	25.97	Interpersonal relationship	19.11	Income	32.75
	Interpersonal relationship	13.51	Interpersonal relationship	17.9	Interpersonal relationship	14.17	Income	15.86	Interpersonal relationship	9.81
	Gender	8.13			Education	4.65	Gender	10.56	Gender	6.59
	Education	5.92					Education	5.5	Education	5.71
							Endowment insurance	1.18		
Mental health	ADL	18.36	Income	23.39	ADL	34.42	Interpersonal relationship	21.03	ADL	28.07
	Gender	17.17	Marriage	22.11	Income	18.47	Gender	20.71	Income	22.55
	Income	16.74	Household registration	19.28	Household registration	13.28	Income	19.7	Gender	21.56
	Household registration	12.33	Interpersonal relationship	16.1	Interpersonal relationship	13.18	ADL	13.41	Interpersonal relationship	9.66
	Interpersonal relationship	11.32	ADL	10.77	Education	13.03	Marriage	13.31	Marriage	8.56
	Education	10.9	Education	6.73	Financial support by children	4.09	Education	11.84	Education	8.22
	Marriage	8.88	Age	1.62	Marriage	3.53			Age	1.38
	Frequency of communication with children	3.04								
	Age	1.26								

The explanatory variables are sorted by contribution rate. The unit of value is percent.

The gender differences in the contribution of each fact were observed further. For life satisfaction and self-rated health, the primary determining factors for male and female older adults are identical. Good income status and interpersonal relationships considerably contribute to life satisfaction. For formal support, only the life satisfaction among male older adults is affected by endowment insurance and medical insurance, which account for 4.56% of the total. The ADL, income, and interpersonal relationships have a greater influence on the self-rated health of male and female older adults. In terms of mental state, there is a significant difference between male and female older adults. For male older adults, income is the most crucial determinant in maintaining mental health, followed by having a spouse or partner and an urban household registration. But having a spouse or partner has a negligible effect on improving female older adults' mental health. Better ADL is critical for preserving the mental health of female older adults, followed by income and urban household registration. However, the effect of ADL on the alleviation of depressive degree in male older adults is just 10.77%.

In addition, the contribution rate of explanatory variables differs between urban and rural areas. Life satisfaction of urban and rural older adults can be improved by focusing on their income status and interpersonal relationships. In terms of formal social support, the impact of endowment insurance and medical insurance on life satisfaction remains modest. Except for interpersonal relationships, the other two variables of informal social support have a relatively minor impact on the life satisfaction of rural older adults. For self-rated health, the ADL continues to have the greatest impact on improving health status. The contribution rate of interpersonal relationships is somewhat higher than income for the health of urban older adults, and the opposite is true for rural older adults' health. Endowment insurance has little influence on the health of urban older adults. Regarding mental state, the contribution rate of influencing factors between urban and rural older adults is quite different. Interpersonal relationships significantly impact the mental state of urban older adults, while the influence on the mental state of rural older adults is only fourth in importance. ADL has a significant impact on the mental state of rural older adults but a much smaller impact on the mental health of urban older adults.

## Conclusion

Based on data from the 2020 CFPS, this article analyzes the impact of social support on quality of life among older adults and determines the contribution of factors to quality of life. Overall, ADL and income improve quality of life considerably. Income plays a significant role in improving life satisfaction, and ADL has the greatest impact on self-rated health and mental state. Both formal and informal social support positively influenced the improvement of the quality of life among older adults, although the contribution of informal social support was greater than that of formal social support. Only life satisfaction is affected by endowment insurance and medical insurance, but their contribution is small. Interpersonal relationships substantially positively affect all three aspects of quality of life.

There are differences in the quality of life among different groups of older adults. For formal social support, endowment and medical insurance play a significant role in increasing life satisfaction among male older adults but have no effect on female older adults. Education level positively affects the self-rated health of female older adults but does not affect male older adults. Male older adults with a spouse or partner have a better mental state. But marital status has a minor effect on the mental state of female older adults. ADL has the most significant impact on the mental state of female older adults but has no discernible impact on the mental health of male older adults. Regarding life satisfaction differences between urban and rural areas, urban older adults are more concerned with endowment insurance, whereas rural older adults pay more attention to medical insurance. Informal social support has a more considerable effect on the life satisfaction of rural older adults than formal social support. Endowment insurance has a slight effect on improving the health status of older adults in urban areas but has no effect on rural older adults. Interpersonal relationships can considerably improve the mental state of urban older adults, while ADL can significantly improve the mental health of rural older adults.

This article attempts to make some suggestions to help improve the quality of life among older adults and the old age security system based on the above findings. The income status considerably impacts all three dimensions of quality of life. Older adults will lose their primary source of income after retirement, diminishing their quality of life. At the national level, formal social support should be strengthened to improve the basic endowment and medical insurance systems. Faced with a massive pension shortfall, the government can delay the retirement age, improve the coverage of enterprise annuities, and establish the third pillar of pension insurance, among other measures. The government should also strengthen the implementation of the basic medical insurance policy, implement cross-provincial direct payment of outpatient fees, broaden the area of medical reimbursement for chronic diseases, and reduce the burden of medicines for older adults. At the family level, the community encourages family members to fulfill their support commitments and increases the willingness of children to support older adults financially. Older adults should be encouraged to re-enter the workforce, demonstrate their social value, and expand their sources of income.

Interpersonal relationships in informal social support had a favorable effect on all three aspects of quality of life, indicating that establishing a good social circle among older adults significantly improved quality of life. Positive interpersonal relationships are fostered through engaging in social activities, such as community governance and entertainment. Older adults should also be instructed and assisted in using intelligent devices and the Internet to expand communication channels and objects. Children's emotional support is also vital. Numerous older adults do not live with their children and live alone, which is detrimental to their physical and emotional health. Children should frequently visit or communicate with older adults through phone or video, be concerned with older adults' living situations, and provide the necessary nursing and financial support.

The impact of social support on quality of life varied among different groups of older adults. Male older adults outperformed female older adults in terms of life satisfaction, self-rated health, and mental state. Therefore, it is important to increase the participation of female older adults in endowment and medical insurance and reduce the burden of family and intergenerational care for female older adults. The policies and services for older adults in urban areas are gradually improving. More attention should be devoted to the spiritual life of urban older adults, and leisure activities should be increased, such as supporting cultural performances and boosting exercise facilities for older adults. It is necessary to expand the coverage of basic endowment and basic medical insurance for rural older adults, raise the level of pension and medical insurance reimbursement standards, and establish a health service and social security system covering rural areas.

There are still some limitations in this article. To ensure the completeness of the data, this article directly deletes samples with missing variable values and does not use interpolation to make up for the data, resulting in a relatively small sample size. This article only analyses the CFPS data in 2020, without considering the survey data of other years, and cannot obtain the dynamic changes of the impact of social support on quality of life. Lastly, this article uses ADL and income status as control variables to determine a direct impact on quality of life and does not consider how both factors affect the correlation between social support and quality of life. This moderating effect will be examined in future research.

## Data availability statement

Publicly available datasets were analyzed in this study. This data can be found here: http://www.isss.pku.edu.cn/cfps/.

## Author contributions

TS drafted the article and conducted the data interpretation. DL conceptualized and designed the research. ZH revised and polished this article. JL and XW collected data and obtained preliminary data results. All authors contributed to the article and approved the submitted version.

## Funding

This work was supported by the Key Program of the National Philosophy and Social Science Foundation of China (Grant No. 21ATJ003), the Innovation Team of Philosophy and Social Sciences in Henan Colleges and Universities (2017–CXTD-07), the 2021 Annual Program of Huamao Financial Research Institute of Henan University of Economics and Law (Measurement and Demonstration of Welfare Based on High- Quality Development), and the National Natural Science Foundation of P.R. China (Grant No. E1190301).

## Conflict of interest

The authors declare that the research was conducted in the absence of any commercial or financial relationships that could be construed as a potential conflict of interest.

## Publisher's note

All claims expressed in this article are solely those of the authors and do not necessarily represent those of their affiliated organizations, or those of the publisher, the editors and the reviewers. Any product that may be evaluated in this article, or claim that may be made by its manufacturer, is not guaranteed or endorsed by the publisher.

## References

[1] Li JX. Social Support and quality of life of the older people in China. Pop Res. (2007) 3:50–60. Available online at: https://kns.cnki.net/kcms/detail/detail.aspx?FileName=RKYZ200703005&DbName=CJFQ2007

[2] National Bureau of Statistics of China. The Seventh National Population Census Report. (2021). Available online at: http://www.stats.gov.cn/tjsj/tjgb/rkpcgb/qgrkpcgb/t20210628_1818824.htm (accessed May 30, 2022).

[3] National Bureau of Statistics of China. The Sixth National Population Census Report. (2011). Available online at: http://www.stats.gov.cn/tjsj/pcsj/rkpc/6rp/html/fu03.htm (accessed May 30, 2022).

[4] Li JW Ji WQ Qian C. The development trend of China's deep aging and demand for older people care services. Reform. (2022) 2:1–21. 10.1186/s12912-022-00809-1 35042481 PMC8767722

[5] State Council. A Guideline to Promote the Development of National Undertakings for the Aged Improve the Older People Care Service System During the 14th Five-Year Plan Period. (2021). Available online at: http://www.gov.cn/zhengce/content/2022-02/21/content_5674844.htm (accessed June 5, 2022).

[6] Galbraith JK. The Affluent Society. Boston, MA: Houghton Mifflin Harcourt (1998).

[7] Asian Development Bank. Asian Development Outlook 1990. Manila: Asian Development Bank (1990). 20 p.

[8] Lin N Wang L Pan YK Yuan GH. The structure and index of quality of life: an analysis of 1985 Survey for 1000 households in Tianjin. Sociol Stud. (1987)6:73–89.

[9] Sarvimäki A Stenbock-Hult B. Quality of life in old age described as a sense of well-being, meaning and value. J Adv Nurs. (2000) 32:1025–33. 10.1046/j.1365-2648.2000.01568.x 11095244

[10] Lu SH Wei LY. Research on the mechanism of subjective and objective indicators of quality of life. Soc Sci China. (1992) 1:121–36.

[11] Feng LT. Research on the quality of life of China's population: progress and interprovincial comparison of the goal of quality of well-off life. Pop Econ. (1995) 6:3–15.

[12] Department of Epidemiology, Beijing Institute of Gerontology, Ministry of Health. Recommendations on survey content and evaluation criteria for quality of life of the elderly (draft). Chin J Geriatr. (1996)15:320.

[13] Wu CP. Improving scientific understanding of the quality of life of the elderly. Pop Res. (2002) 5:1–5. Available online at: https://kns.cnki.net/kcms/detail/detail.aspx?FileName=RKYZ200205000&DbName=CJFQ2002

[14] Van Leeuwen KM Van Loon MS Van Nes FA Bosmans JE De Vet HC Ket JC. What does quality of life mean to older adults? A thematic synthesis. PLoS ONE. (2019) 14:e0213263. 10.1371/journal.pone.0213263 30849098 PMC6407786

[15] Cobb S. Social support as a moderator of life stress. Psychosom Med. (1976) 38:300–14. 10.1097/00006842-197609000-00003 981490

[16] Tao YC Shen Y. The influence of social support on the physical and mental health of the rural elderly. Pop Econ. (2014) 3:3–14. Available online at: https://kns.cnki.net/kcms/detail/detail.aspx?FileName=RKJJ201403002&DbName=CJFQ2014

[17] Flannery RB. Social support and psychological trauma: a methodological review. J Trauma Stress. (1990) 3:593–611. 10.1002/jts.2490030409

[18] Mindel CH Wright Jr R Starrett RA. Informal and formal health and social support systems of black and white elderly: a comparative cost approach. Gerontologist. (1986) 26:279–85. 10.1093/geront/26.3.279 3087823

[19] Zhang C Han H. Urban-rural differences: the impact of social support on the use of multiple healthcare services for older people. Front Public Health. (2022) 10:851616. 10.3389/fpubh.2022.851616 35493353 PMC9051021

[20] Krause N. Satisfaction with social support and self-rated health in older adults. Gerontologist. (1987) 27:301–8. 10.1093/geront/27.3.301 3609798

[21] Berkman LF. Assessing the physical health effects of social networks and social support. Annu Rev Public Health. (1984) 5:413–32. 10.1146/annurev.pu.05.050184.002213 6372817

[22] Berkman LF. Social networks, support, and health: taking the next step forward. Am J Epidemiol. (1986) 123:559–62. 10.1093/oxfordjournals.aje.a114276 3513547

[23] He ZP. Socioeconomic status and social support network of the rural elderly and their physical and mental health. Soc Sci China. (2002) 3:135–48+207. Available online at: https://kns.cnki.net/kcms/detail/detail.aspx?FileName=ZSHK200203011&DbName=CJFQ200229866373

[24] Xiang YH Yao H. The urban-rural difference of social support for the aged and its impact on their health situation and life satisfaction. J Huazhong Agric Univ. (2016) 6:85–92+145. 10.13300/j.cnki.hnwkxb.2016.06.012

[25] Zheng ZD Zheng YH. The influence of social support on the health and life satisfaction of the elderly: reexamine based on the endogenous of intergenerational economic support. Pop Econ. (2017) 4:63–76. Available online at: https://kns.cnki.net/kcms/detail/detail.aspx?FileName=RKJJ201704007&DbName=CJFQ2017

[26] Deng DS Tang JL. Life satisfaction of the elderly and its influencing factors: based on CHARLS2018 data. Theory Monthly. (2021) 12:116–24. 10.14180/j.cnki.1004-0544.2021.12.013

[27] Li D Li X Zeng Y. The moderating effect of community environment on the association between social support and chinese older adults' health: an empirical analysis study. Front Public Health. (2022) 10:855310. 10.3389/fpubh.2022.855310 35570963 PMC9092342

[28] Wei Y Liu XD Zhang YP. The influence of social support on the physical and mental health of the rural elderly. Pop J. (2010) 4:41–7. Available online at: https://kns.cnki.net/kcms/detail/detail.aspx?FileName=RKXK201004007&DbName=CJFQ2010

[29] Li M. Effects of Social Support on Quality of Life Among the Elderly in China. Southwestern University of Finance and Economics (2019). 10.27412/d.cnki.gxncu.2019.002002

[30] Fu X Cheng ZY. Research on the quality of life difference of the elderly in china: an empirical analysis based on CFPSIntertemporal Data. Northwest Pop J. (2021) 42:10–25. 10.15884/j.cnki.issn.1007-0672.2021.01.00231817811

[31] Liu YL Wang L Zhao Q. Settlement of index system on the aged life of quality. J Chongqing Univ. (2005) 8:154–8. Available online at: https://kns.cnki.net/kcms/detail/detail.aspx?FileName=FIVE200508039&DbName=CJFQ2005

[32] Li D Li SL. Impacts of caring for parents on adult children's health wellbeing: an empirical analysis based on Data from CFPS (2016). Sci Res Aging. (2021) 9:63–78. Available online at: https://kns.cnki.net/kcms/detail/detail.aspx?FileName=LLKX202110008&DbName=CJFQ2021

[33] Shorrocks AF. Decomposition procedure for distributional analysis: a unified framework based on the Shapley value. J Econ Inequality. (2013) 11:99–126. 10.1007/s10888-011-9214-z

